# CCDC80 suppresses high‐grade serous ovarian cancer migration via negative regulation of B7‐H3

**DOI:** 10.1002/1878-0261.70235

**Published:** 2026-04-21

**Authors:** Aya Saleh, Nitzan Medina‐Itzhaki, Milena Chekov, Eden Gal‐Swisa, Roba Gabesh‐Wahabi, Inbar Savyon, Inna Naroditsky, Hilary A. Kenny, Basem Fares, Lina Korsensky, Einav Girsh, Liron Berger, Ruth Perets

**Affiliations:** ^1^ Division of Oncology, Clinical Research Institute at Rambam Rambam Health Care Campus Haifa Israel; ^2^ Women's Cancers Research Lab, Ruth and Bruce Rappaport Faculty of Medicine Technion ‐ Israel Institute of Technology Haifa Israel; ^3^ Department of Pathology Rambam Health Care Campus Haifa Israel; ^4^ Department of Obstetrics and Gynecology/Section of Gynecologic Oncology The University of Chicago Illinois USA

**Keywords:** B7‐H3, CCDC80, Ovarian cancer, PAX8, Tumor suppressor, Migration

## Abstract

Paired box protein Pax‐8 (*PAX8*) is a critical lineage marker and master regulator of transcription in high‐grade serous ovarian carcinoma (HGSC)—the most common subtype of epithelial ovarian cancer—driving cell proliferation and migration and resisting apoptosis. This study aimed to elucidate the mechanism of action of PAX8 in this disease. By performing an unbiased analysis of PAX8‐regulated genes, we discovered two PAX8‐regulated genes—coiled‐coil domain‐containing protein 80 (*CCDC80*) and cluster of differentiation 276 (*CD276*) antigen (also known as B7‐H3)—that mediate PAX8 activity and play key cancer cell autonomous roles in this disease. Our findings indicate that PAX8 negatively regulates *CCDC80*, a novel cell autonomous tumor suppressor in HGSC. We demonstrate that CCDC80, localized to the nucleus, significantly reduces HGSC tumor growth and metastasis *in vivo* in a mouse model. Notably, CCDC80 exerts its function by suppressing the expression of the immune checkpoint protein B7‐H3. However, in HGSC, B7‐H3 is predominantly cytoplasmic and promotes HGSC proliferation and migration independent of its immune role. Additionally, PAX8 positively regulates *B7‐H3* expression in a CCDC80‐independent manner, underscoring the multifaceted oncogenic role of PAX8. This study highlights the complex regulatory network involving PAX8, CCDC80, and B7‐H3 in HGSC progression. Targeting this signaling pathway may provide a novel therapeutic strategy to improve treatment outcomes for patients with epithelial ovarian cancer. B7‐H3, which is currently targeted in clinical trials, shows promise as HGSC target for therapy.

## Graphical abstract

PAX8 is a lineage‐specific master regulator of transcription in high‐grade serous ovarian cancer (HGSC) progression. We show for the first time that PAX8 facilitates proliferation and metastasis by repressing the cell autonomous tumor suppressor CCDC80 and inducing B7‐H3 expression. These newly identified mediators of HGSC metastatic potential reveal novel opportunities for therapeutic intervention.

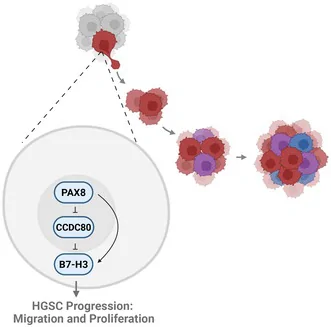

**Abbreviations**
B7‐H3: B7 Homolog 3
BSA: bovine serum albumin
*CCDC80*
: coiled‐coil domain containing 80
*CD276*
: cluster of differentiation 276
ChIP: chromatin immunoprecipitation
CTGF: Connective Tissue Growth Factor
DMEM: Dulbecco's modified Eagle's medium
ECL: enhanced chemiluminescence
ENCODE: Encyclopedia of DNA Elements
EOC: epithelial ovarian cancer
FBS: fetal bovine serum
FDR: false discovery rate
FGF18: fibroblast growth factor 8
FLT3: FMS‐like tyrosine kinase 3
FTSEC: fallopian tube secretory epithelial cells
GAPDH: glyceraldehyde‐3‐phosphate dehydrogenase
H&E: hematoxylin and eosin
H3K27Ac: histone H3 lysine 27 acetylation
HGSC: high‐grade serous ovarian carcinoma
hr: hour
HRP: horseradish peroxidase
IACUC: Institutional Animal Care Use Committee
IF: immunofluorescence
IHC: immunohistochemistry
KD: knockdown
LFQ: label‐free quantification
LMO3: LIM domain only 3
MITF: microphthalmia‐associated transcription factor
mRNA: messenger RNA
MTS: 3‐(4,5‐dimethylthiazol‐2‐yl)‐5‐(3‐carboxymethoxyphenyl)‐2‐(4‐sulfophenyl)‐2H tetrazolium
NT‐siRNA: nontargeting small interfering RNA
PAGE: polyacrylamide gel electrophoresis
PARP: poly (ADP‐ribose) polymerase
PAX8: paired box protein 8
PBS: phosphate‐buffered saline
PCR: polymerase chain reaction
qPCR: quantitative PCR
RIPA: radioimmunoprecipitation assay
RT: room temperature
S100A1: S100 calcium binding protein A1
SDS: sodium dodecyl sulfate
SHMT1: serine hydroxy methyltransferase 1
TBP: TATA box binding protein
TCGA: The Cancer Genome Atlas
TMA: tissue micro‐array
TPM1: tropomyosin 1
USPC: uterine serous papillary cancer
UTR: untranslated region

## 1 Introduction

Epithelial ovarian cancer (EOC) is the deadliest gynecological malignancy and the 6th most common cause of cancer death in the western world [1], primarily due to a combination of late diagnosis and lack of good treatment options for advanced disease [2]. Immunotherapy has largely been ineffective in this setting, with chemotherapy and surgery remaining the cornerstone of treatment [3]. In recent years, some progress has been made with the approval of several new targeted therapies [4, 5, 6] reinforcing the role of targeted treatments as an emerging modality for epithelial ovarian cancer. This progress highlights the need for better understanding of targetable mechanisms driving epithelial ovarian cancer progression and metastasis to develop more effective targeted therapies.

Multiple studies have established that high‐grade serous ovarian cancer (HGSC), the most common subtype of epithelial ovarian cancer, arises in most cases from the fallopian tube epithelium, rather than from the ovary [2, 7, 8], raising an interest in fallopian tube lineage markers. The most prominent fallopian tube lineage marker is PAX8, a transcription factor involved in the embryonic development of the female genital tract [9, 10]. In adult women, PAX8 is expressed in fallopian tube secretory epithelial cells (FTSEC), the proposed cells of origin of HGSC [8, 11]. During transformation of FTSEC to HGSC, PAX8 expression is retained and it is ubiquitously expressed in nearly all HGSC tumors [12, 13]. PAX8 has been identified as a master transcriptional regulator in HGSC, modulating key oncogenic processes such as evading apoptosis [14, 15], cell migration [16], and angiogenesis [17]. Despite its clear protumorigenic roles, PAX8 is not classified as an oncogene, as its expression remains unaltered in most HGSC tumors, failing to meet the criteria for oncogenes. *PAX8* amplification occurs in only 16% of HGSC cases, and it is unclear whether its expression is consistently upregulated compared to benign FTSEC cells. Moreover, *PAX8* mutations have not been detected [18]. Instead, PAX8 serves as a marker of fallopian tube lineage, becoming critical during the transformation process. How PAX8 becomes tumorigenic during transformation is unclear, but likely the protumorigenic potential is mediated by changes in the PAX8 transcriptome [19], rather than changes in PAX8 itself.

In this study, we identify two novel PAX8‐regulated target genes, both of which play a role in cancer–microenvironment interactions and demonstrate a previously unrecognized cell‐autonomous function for each that is independent of the tumor microenvironment. These genes are *coiled‐coil domain containing 80* (*CCDC80*), encoding the protein of the same name, and *cluster of differentiation 276* (*CD276*), which encodes the B7‐H3 protein. CCDC80 is primarily known for its role in glucose homeostasis in diet‐induced obese mice [20, 21]. There is limited evidence suggesting a tumor‐suppressive role for CCDC80, including in ovarian cancer [22], although this role was attributed to its expression in fibroblasts rather than cancer cells [23]. While CCDC80 expression is reduced in ovarian cancer tumors as compared to other tissues [24], it is unclear whether this reduced expression stems from fibroblasts or epithelial cancer cells. Our findings reveal a tumor suppressive role for CCDC80 expressed in cancer cells. B7‐H3 is well recognized for its role in immune regulation [25], and here we show, similar to CCDC80, that B7‐H3 also exhibits a cell‐autonomous role in promoting ovarian cancer progression.

## 2 Materials and methods

### 2.1 Cell culture and transfection reagents

OVCAR4 (RRID:CVCL 1627) and OVCAR8 (RRID:CVCL_1629) cells were cultured in DMEM/F12 HAM (1 : 1) (Sartorius AG, Göttingen, Germany) supplemented with 10% fetal bovine serum (FBS, Sigma Aldrich, St. Louis Missouri). OVCAR3 (RRID:CVCL_0465) cells were cultured in DMEM/F12 HAM supplemented with 15% FBS. Kuramochi (RRID:CVCL_1345) cells were cultured in RPMI medium (Sartorius AG) with 10% FBS, and HEK293FT (RRID:CVCL_6911) cells in DMEM (high glucose) containing 10% FBS, 1% L‐glutamine, 1% sodium pyruvate, and 1% nonessential amino acids. All cell lines were a kind gift from Prof. Ronny Drapkin (U Penn) and were regularly tested and found negative for mycoplasma contamination. Also, the source of the cell lines was authenticated by sequencing p53 to assure they carry the expected mutations. Lipofectamine™ RNAiMAX transfection reagent (Invitrogen, Thermo Fisher Scientific Corp., Waltham, MA, USA) was used in knockdown experiments, and X‐tremeGENE™ HP DNA transfection reagent (F. Hoffmann‐La Roche AG, Basel, Switzerland) in overexpression experiments. Opti‐MEM serum‐reduced medium (Gibco, Thermo Fisher Scientific Corp., Waltham, MA, USA) was used for transfection.

### 2.2 Knockdown and overexpression experiments

*PAX8‐1* and *PAX8‐2* siRNA constructs were purchased from Dharmacon™ (Lafayette, Colorado) as On‐Target plus siRNA collection (cat. no. J‐003778‐07 and J‐003778‐10, respectively). *CCDC80* siRNA was purchased as SMARTpool™ (Dharmacon™, cat no. L‐018258‐02‐0005). *B7‐H3‐1* and *B7‐H3‐2* siRNA constructs were purchased as Silencer® Select siRNA sequences s37288 and s37290 (ThermoFisher, Cat. no. 4392420). As a negative control for siRNA transfection, we used On‐Target plus non‐targeting siRNA (NT‐siRNA, Dharmacon™, cat. no. D‐001810‐02‐05). For human CCDC80 overexpression, we used a human full‐length *CCDC80* coding sequence that was cloned into a pcDNA3.1+ vector and synthesized by GenScript. pcDNA3.1+ vector was used as a negative control and was a kind gift from Prof. Raz Palty (Technion—Israel Institute of Technology, Haifa, Israel).

### 2.3 Generation of stable CCDC80 overexpressing HGSC cell lines

The human full‐length *CCDC80* coding sequence was subcloned in the BamHI‐XhoI site of the viral plasmid p170_NSPI‐CMV_MCS_myc_His (a kind gift from Haguy Wolfenson's lab, Technion, Israel). The resulting plasmids were sequenced to validate *CCDC80* insertion into the viral plasmid. Lentiviral particles were produced from HEK293FT cells using the packaging plasmid psPAX2 (addgene#12260) and the envelope plasmid PMD2G (addgene#12259). Viral particles were collected as previously described [26] and were used to infect OVCAR3 and OVCAR4 cells. CCDC80 overexpressing cells and their controls were selected using Puromycin.

### 2.4 Proliferation assay

Cells were seeded onto 96‐well plates in six replicates. For transient transfections, three to 7 days after the transfection, cell proliferation was measured using an MTS‐based kit (Promega CellTiter 96™), and the absorbance was measured using a BioTek microplate reader. In other experiments, stable CCDC80‐overexpressing HGSC cells and their controls were seeded in 96‐well plates, and cell proliferation was analyzed at Day 3 and normalized to Day 0. The results are presented as relative cell number compared to control cells. P‐value was calculated using a two‐tailed unpaired Student's *t*‐test.

### 2.5 Colony formation assay

To measure clonogenic capacity, cells were seeded in 12‐well plates, transfected 24 h later (unless stably expressing cells were used), and twenty‐four hours later re‐plated in 6‐well plates at low density (1000–2000 cells/well) in triplicates. The cells were allowed to grow and form colonies for 10–15 days. Then, cells were fixed with 4% formaldehyde for 10 min, stained with 1% crystal violet solution (Sigma Aldrich, St. Louis, MO, USA; cat. no. V5265), washed with water, and dried. The number of colonies in photographed wells was counted using the ImageJ software. Representative images are shown. All three wells were quantified, and the average number of colonies was used for further analysis. Each figure represents data from three independent experiments. *P*‐value was calculated using a two‐tailed paired Student's *t*‐test.

### 2.6 Wound healing assay

To measure cancer cell migration *in vitro*, cells were seeded in ImageLock 96‐well plates (Sartorius AG, Göttingen, Germany) at 30‐60 × 10^3^ cells per well to obtain 100% monolayer confluence on the day of transfection. A transient transfection was performed as described above 24 h later. Then, a scratch was generated by a Woundmaker Tool (Sartorius AG, Göttingen, Germany), and the plates were incubated for 1–3 days in an IncuCyte ZOOM™ real‐time live cell imaging system (Essen BioScience, Ann Arbor, MI, USA). Wound width was measured every 1–2 h. All wound healing assays were done with at least eight replicates. *P*‐value was calculated using two‐tailed paired Student's *t*‐test.

### 2.7 *In vivo* cell migration assay

*In vivo* cell migration assay was adapted from [27]. For *in vivo* cell migration experiments, CCDC80‐overexpressing and control OVCAR4 cells were stained with CellTracker™ Red CMTPX fluorescent dye (ThermoFisher, cat. no. C34552) and 5 × 10^6^ cells were injected intraperitoneally into 6 weeks‐old female Hsd:Athymic‐Nude‐Foxn1‐nu mice (Envigo, Israel, *n* = 5/group). 24 h later, mice were sacrificed, and the omentum was removed and collected in phosphate‐buffered saline (PBS). Fluorescent images of the omentum were taken using Olympus Stereoscope microscope prior to incubation in 5% NP‐40 for lysis. Lysates from the omentum were collected to a flat black 24‐well plate (Cellvis, cat. P24‐1.5H‐N) for fluorescence intensity measurement using Plate Reader Infinite M200 PRO (Tecan, Männedorf, Switzerland). Mice were maintained under specific‐pathogen‐free (SPF) conditions on a 12‐h light: 12‐h dark cycle and housed in groups of 2–5 mice per cage, with food and water *ad libitum*. These experiments were approved by the Technion Institutional Animal Care Use Committee (IACUC) approval number IL‐127‐07‐2023 and performed according to the National Institutes of Health guidelines and relevant ethical regulations.

### 2.8 *In vivo* xenograft model

For xenograft experiments, CCDC80‐overexpressing and control OVCAR3 cells were harvested and resuspended in PBS. A total of 5 × 10^6^ cells were injected subcutaneously into the right flanks of 8‐week‐old female Nude‐Foxn1‐nu mice (Envigo, *n* = 7/group). Tumor growth and general health status were monitored weekly. After 16 weeks, the mice were sacrificed and tumors were excised, counted, and H&E staining was performed. Mice were maintained under specific‐pathogen‐free (SPF) conditions on a 12‐h light: 12‐h dark cycle and housed in groups of 2–5 mice per cage, with food and water *ad libitum*. These experiments were approved by the Technion Institutional Animal Care Use Committee (IACUC) approval number IL‐127‐07‐2023 and performed according to the National Institutes of Health guidelines and relevant ethical regulations.

### 2.9 Immunohistochemistry

Histological human samples of high‐grade serous ovarian tumors were obtained from the Department of Pathology at the Rambam Health Care Campus (Ethics approval number 0426‐14‐RMB). Samples were collected between July 2023 to March 2024, diagnosed and confirmed by a senior pathologist (I.N.). This work was conducted in accordance with the guidelines of the Declaration of Helsinki. Due to the retrospective nature of sample collection, informed consent was waived by the local institutional ethics committee under the approved study protocol. Paraffin‐embedded sections (4 μm) were cut on coated slides, deparaffinized, and rehydrated. In addition, a tissue micro‐array (TMA) was purchased from TissueArray.com (OV244), containing seven cases of HGSC, and controls. Antigen retrieval was performed using Citrate‐based Antigen Unmasking Solution (pH 6.0, Vector Laboratories, Inc., cat. no. H‐3300) in a microwave for 20 min, followed by blocking with 5%BSA in PBS for 30 min at room temperature (RT), and permeabilization with 0.2% Triton X‐100 in PBS for 10 min. Endogenous peroxidase activity was neutralized using a hydrogen peroxidase block buffer (Abcam, Cambridge, UK; cat. no. Ab64260), and slides were incubated overnight at 4 °C with B7‐H3 primary antibody (1 : 100, Abcam, cat. no. ab227670) in 1%BSA. Rabbit‐specific HRP/AEC detection IHC kit (Abcam cat. no. ab64260) was used for signal detection and AEC was used as a substrate. Slides were then counterstained with hematoxylin, washed with distilled water and mounted. Images were taken using a standard light microscope or scanned by automatic slide scanner 250 Flash and viewed by the SlideViewer software.

### 2.10 Chromatin immunoprecipitation (ChIP)

ChIP experiments were adapted from a previously published protocol [28], and all ChIP reactions were performed in three independent replicates. Briefly, ten million Kuramochi HGSC cells were cross‐linked with 1% formaldehyde for 10 min, and then cross‐linking was stopped using glycine. Cells were scraped, washed in PBS, and cell nuclei were isolated using a series of washes and centrifugations in lysis buffers 1, 2 and 3 (all lysis buffers are taken from [28] and used with protease inhibitors). Nuclei lysates were then sonicated using medium power and eight cycles of 30 s on 40 s off incubated with antibodies that were pre‐incubated with Dynabeads magnetic beads. For PAX8, we used 10 μg of Novus cat# NBP1‐32440 antibody, and for H3K27Ac, we used 3 μg of Diagenode cat #C15410196. Beads were then washed with RIPA buffer and LiCl buffer and Tris‐EDTA buffer, and then eluted with an elution buffer as previously described [28]. RNase, proteinase K and glycogen, were added to the elution buffer and the eluted fraction was incubated at 65 °C for 16 h. Then, the DNA was purified using a phenol‐chloroform and ethanol precipitation, and sent for sequencing together with an input sample, to the Technion Genome Center. Sequencing was performed on an Illumina HiSeq 2500 machine, followed by a quality control pipeline. 36–48 million reads were found for each sample, of which 2% were unknown reads. The quality of the run was determined using FASTQC version 0.11.5. Peak calling was performed using MACS2 v2.1.1. Sequences were aligned using BWA v. 0.7.17 with Homo_Sapiens.GRCh38 as the reference genome. Estimated fragment length was 240–300 bp and enriched peaks were considered if they passed the FDR threshold of 0.05.

### 2.11 Western blot analysis

Western blot analysis was performed as previously described [26]. In brief, cells were lysed for 20 min on ice to extract the total protein using homemade RIPA buffer (Tris–HCl 50 mm, EDTA 1 mm, NaCl 150 mm, Triton X‐100 1%, SDS 0.1%, Na‐Doc 0.5%) with complete protease inhibitor cocktail (Roche, cat. no. 11836153001) and subsequently centrifuged (14 000 ***g*** for 20 min at 4 °C). Total protein was quantified using Bradford protein assay (Bio‐Rad Laboratories Inc., Hercules, CA, USA). The extracted protein was mixed with sample buffer containing β‐mercaptoethanol and boiled for 5 min at 95 °C for protein denaturation. Total protein (10‐70 μg) was loaded on 8–12% SDS/PAGE gel and the separated proteins were subsequently transferred onto a nitrocellulose membrane (Bio‐Rad, cat. no. 1704158). After blocking with 5% BSA (Sigma Aldrich, Rehovot, Israel) in Tris‐buffered saline containing 0.1% Tween‐20 (TTBS) for 1 h at room temperature, the membranes were incubated with primary antibodies diluted in 5% BSA, washed three times with TTBS buffer, and incubated with a horseradish peroxidase (HRP)‐conjugated secondary antibody diluted in TTBS buffer for 1 h at room temperature. A list of primary and secondary antibodies can be found in Table S1. The HRP‐conjugated antibody was detected using ECL kit (Biological Industries, Beit HaEmek, Israel), and visualized using ImageQuant™ LAS 4000 imaging system.

### 2.12 RNA extraction, cDNA synthesis, and real‐time quantitative PCR

Total RNA was isolated from cells using TRIzol reagent (Ambion Inc., Austin, Texas), according to the manufacturer's instructions. The quantity and purity of RNA were confirmed using a NanoDrop™ 2000c spectrophotometer (Thermo Fisher Scientific Inc.). DNase I (Thermo Scientific™ cat. no. EN0521) treatment was performed and cDNA was synthesized by reverse transcribing 1 μg RNA into cDNA using the High‐Capacity cDNA Reverse Transcription kit (Applied Biosystems, Waltham, MA, USA). The resulting cDNA was diluted at a 1 : 10 proportion with nuclease‐free water and then used as the template for subsequent real‐time quantitative PCRs (qPCR) or stored at −20 °C. Real‐time qPCR was conducted on a QuantStudio3 Real‐Time PCR device (Applied Biosystems) using Fast SYBR® Green Master Mix (Applied Biosystems) according to the manufacturer's instructions. These data were analyzed by the 2^−ΔΔ*C*t^ method where the gene encoding TATA box binding protein (*TBP*) was assigned as the housekeeping gene. The results are presented as relative mRNA expression as compared to the control. Each experiment was performed in biological triplicates and *P*‐value was calculated using a two‐tailed Student's *t*‐test. The primer sequences used are listed in Table S2.

### 2.13 Apoptosis assay

Apoptosis was evaluated by performing western blot analysis for cleaved PARP or cleaved caspase‐3 expression levels.

### 2.14 Immunofluorescence

Cells were seeded on coverslips in 24‐well plates and fixed with 4% paraformaldehyde for 10 min, permeabilized with 0.1% Triton X‐100 (in PBS) for 5 min, blocked with 5% BSA for 1 h at RT and incubated with primary antibodies for CCDC80 (1 : 50, Bioss, cat. no. bs‐7992R) or B7‐H3 (1 : 25, Abcam, cat. no. ab227670) in 5% BSA overnight at 4 °C. After washes, the cells were incubated with donkey anti‐rabbit Rhodamine‐Red‐X conjugated secondary antibody (1 : 200, Jackson ImmunoResearch Laboratories, West Grove, PA, USA; cat. no. 711–295‐152) diluted in 4%BSA and 1% Tween‐20 (in PBS) in the dark for 1 h and counterstained with 1 ng·mL^−1^ DAPI. Cells incubated with secondary antibody only served as a negative control. Slides were visualized using a confocal LSM 700 inverted microscope, and images were captured at 20× and 63× magnification.

### 2.15 Proteolysis and mass spectrometry analysis

The protein pellets were dissolved in 9 m Urea and 400 mm ammonium bicarbonate, sonicated, then reduced with 3 mm DTT (60 °C for 30 min), modified with 10 mm iodoacetamide in 100 mm ammonium bicarbonate (room temperature 30 min in the dark), and digested in 2 M Urea, 25 mm ammonium bicarbonate with modified trypsin (Promega, Madison, WI, USA), overnight at 37 °C in a 1 : 50 (M/M) enzyme‐to‐substrate ratio. The tryptic peptides were desalted using C18 tips (Top tip, Glygen), dried, and re‐suspended in 0.1% formic acid.

The peptides were resolved by reverse‐phase chromatography on 0.075 × 300‐mm fused silica capillaries (J&W) packed with Reprosil reversed phase material (Dr. Maisch GmbH, Germany). The peptides mixture was resolved with a (5 to 28%) linear gradient of solvent B (95% acetonitrile with 0.1% formic acid) for 180 min followed by a gradient of 15 min of 28 to 95% and 15 min at 95% acetonitrile with 0.1% formic acid in water at flow rates of 0.15 μL·min^−1^. Mass spectrometry was performed by Q Exactive HF mass spectrometer (Thermo) in a positive mode (m/z 300–1800, resolution 120 000 for MS^1^ and 15 000 for MS^2^) using repetitively full MS scan followed by collision induced dissociation (HCD, at 27 normalized collision energy) of the 30 most dominant ions (> 1 charges) selected from the first MS scan. The AGC settings were 3 × 10^6^ for the full MS and 1 × 10^5^ for the MS/MS scans. A dynamic exclusion list was enabled with an exclusion duration of 20 s.

The mass spectrometry data were analyzed using the MaxQuant software 1.5.2.8 [29] for peak picking and identification using the Andromeda search engine, searching against the human proteome from the Uniprot database with mass tolerance of 6 ppm for the precursor masses and 20 ppm for the fragment ions. Oxidation on methionine and protein N‐terminal acetylation were accepted as variable modifications, and carbamidomethyl on cysteine was accepted as a static modification. Minimal peptide length was set to six amino acids, and a maximum of two miscleavages was allowed. The data were quantified by label‐free analysis using the same software. Peptide‐ and protein‐level false discovery rates (FDRs) were filtered to 1% using the target‐decoy strategy. The protein table was filtered to eliminate the identifications from the reverse database, common contaminants, and single peptide identifications. Missing values were replaced with 20 (in log_2_), which was the threshold intensity. Statistical analysis of the identification and quantification results was done using Perseus 1.6.10.43 software [30].

## 3 Results

### 3.1 PAX8 negatively regulates *CCDC80* expression in HGSC

**Fig. 1:**
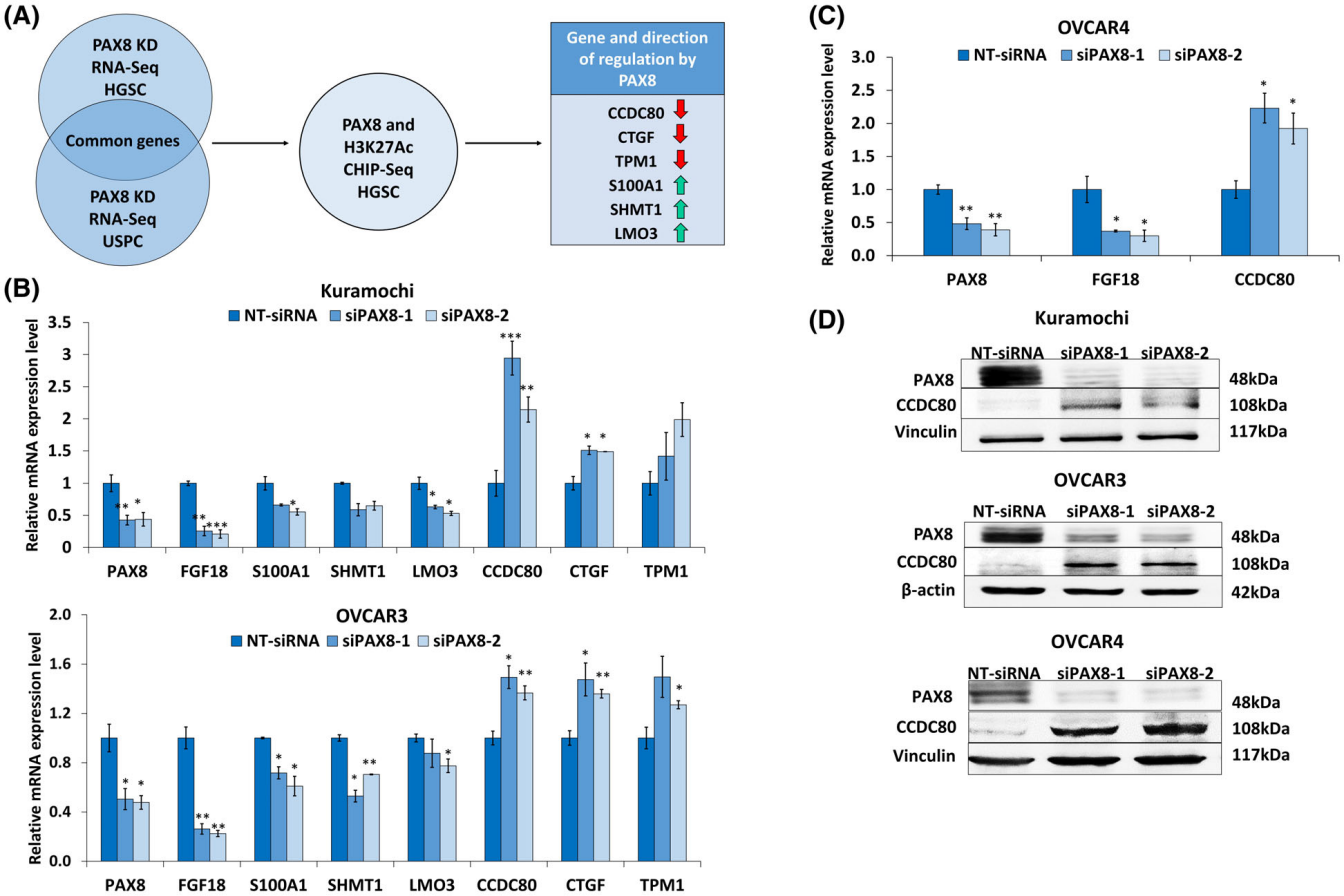
PAX8 negatively regulates CCDC80 expression in high‐grade serous ovarian cancer. (A) An illustration of a bioinformatics analysis. Two publicly available data sets of PAX8 co‐regulated genes were analyzed, one by Elias *et al*. [19], and one by our group [26]. Twenty‐four genes were differentially expressed in at least four of the five lines studied and were crossed with PAX8 bound genes defined by PAX8 ChIP‐Seq analysis performed in Kuramochi (HGSC) cells. This yielded six candidate PAX8 directly regulated genes that are listed in the rectangle. Whether genes are positively or negatively regulated by PAX8 is marked with an arrow. KD, Knockdown, HGSC, high‐grade serous ovarian carcinoma, Chip, chromatin immunoprecipitation, FDR, false discovery rate. (B, C) Kuramochi, OVCAR3 (B) or OVCAR4 (C) cells were transfected with either non‐targeting siRNA (NT‐siRNA) or one of two different *PAX8* siRNA (siPAX8) constructs, and then mRNA expression levels of the mentioned genes were measured using real‐time qPCR, normalized to *TBP*, and shown relative to NT‐siRNA. Data shown represent mean values ± standard error from three independent biological experiments, each performed with three technical replicates per condition. Statistical analysis was performed using two‐tailed Student's *t*‐test comparing siPAX8 to NT‐siRNA. *P < 0.05, **P < 0.01, ***P < 0.001. NT, non‐targeting, *TBP*, *TATA‐box binding protein*. (D) Kuramochi, OVCAR3 and OVCAR4 cells were transfected with NT‐siRNA or siPAX8. Total protein was extracted 48–72 h post transfection and western blot analysis for PAX8 and CCDC80 was performed. Vinculin and β‐actin were used as loading controls. Blots shown are representative of three independent biological experiments.

PAX8 is a key transcriptional master regulator in HGSC [14, 19] and uterine serous papillary cancer (USPC) [26], exerting its oncogenic effects through the transcriptional activation of numerous target genes. To investigate which of these genes contribute to its oncogenic potential, we hypothesized that genes regulated by PAX8 in both HGSC and USPC play a pivotal role in driving its protumorigenic effects. To test this, we analyzed two datasets of *PAX8* coregulated genes. In previous work, we performed RNA sequencing after PAX8 knockdown in two USPC cell lines, USPC‐ARK‐1 and USPC‐ARK‐2 [26]. We then cross‐referenced this dataset with PAX8‐regulated genes from three HGSC cell lines reported by Elias *et al*. [19], namely Kuramochi, OVSAHO and JHOS4. We focused on genes significantly upregulated or downregulated following PAX8 knockdown, with a false discovery rate (FDR) < 0.05, across at least four of the five cell lines studied. Our analysis revealed a list of 24 genes that were differentially regulated by PAX8 in HGSC and UPSC (Fig. 1A and Table S3). Next, we performed PAX8 and H3K27Ac (a marker of active enhancers) chromatin immunoprecipitation (ChIP) in Kuramochi HGSC cells followed by next generation sequencing (ChIP‐Seq) and compared the precipitated fraction to input sequences. Genomic areas in the precipitated fraction as compared to input were calculated using model based analysis of ChiP‐Seq (MACS) [31]. Peaks were connected to genes if they were up to 3Kb upstream to the transcription start site or up to 300‐bp downstream of the 3′UTR. Then the list of PAX8 differentially regulated genes was crossed with the list of PAX8 bound genes and five genes were defined as genes that were differentially regulated by PAX8 in both cancer types and bound by PAX8 (*PAX8, CCDC80, CTGF, TPM1, LMO3*); all but *CTGF* were also bound by H3K27Ac. Two genes from the list of differentially expressed genes were not bound by PAX8 but were in the list of H3K27Ac bound genes and therefore added to the list (*S100A1* and *SHMT1*). The list of genes can be found in Fig. 1A (PAX8 that regulates its own expression was removed from the list). To validate the regulation of these genes by PAX8, we performed real‐time quantitative PCR (qPCR) analysis in Kuramochi and OVCAR3 HGSC cell lines, using FGF18 as a positive control [32] (Fig. 1B). Of the tested genes, *CCDC80* stood out as consistently negatively regulated by PAX8 in HGSC. The negative regulation of *CCDC80* by PAX8 was further validated in OVCAR4 HGSC cells at the mRNA level (Fig. 1C). We have also validated that PAX8 negatively regulates CCDC80 protein expression levels (Fig. 1D).

### 3.2 CCDC80 inhibits proliferation and colony formation of HGSC cells

**Fig. 2:**
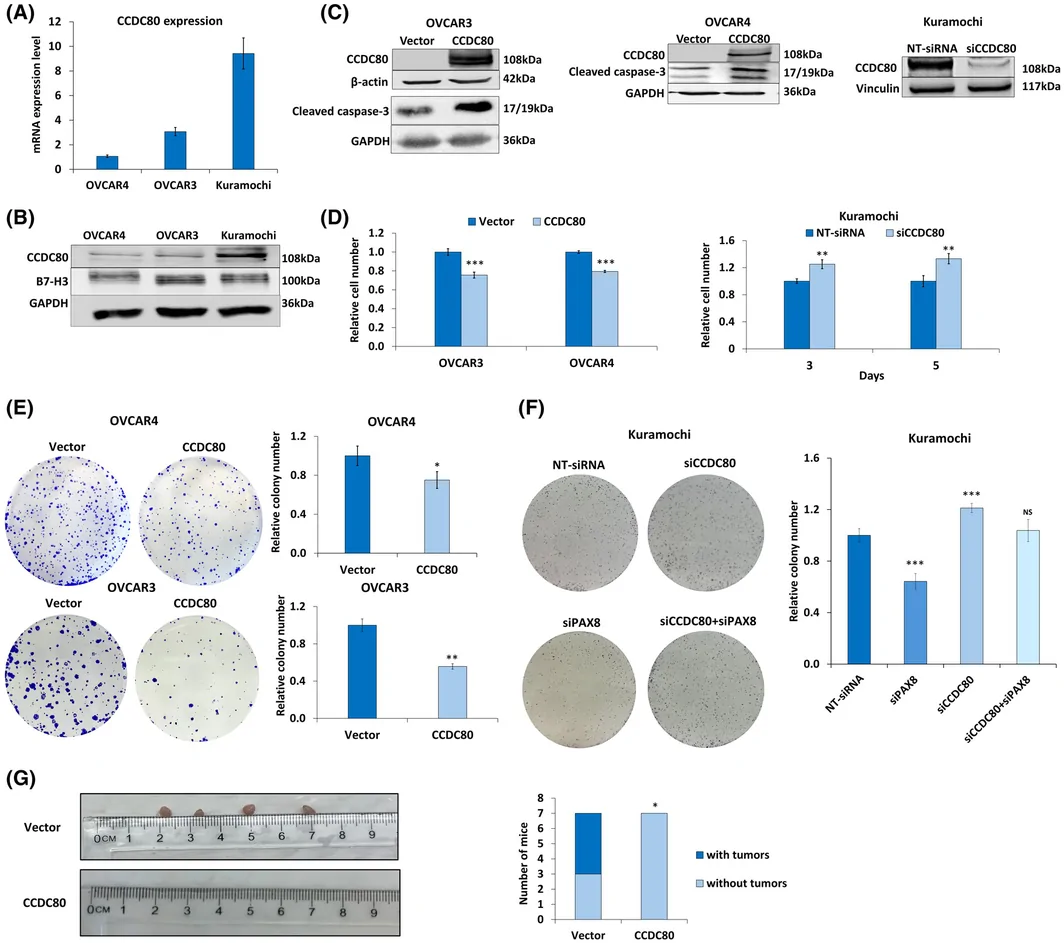
CCDC80 inhibits proliferation and colony formation of high‐grade serous ovarian cancer cells. (A) Real‐time qPCR analysis of *CCDC80* mRNA levels in OVCAR4, OVCAR3 and Kuramochi HGSC cells. mRNA expression levels were normalized to those of *TBP*. Data shown represent mean values ± standard error from three independent experiments. Statistical analysis was performed using a two‐tailed unpaired Student's *t*‐test. HGSC, high‐grade serous ovarian carcinoma, qPCR, quantitative PCR, *TBP*, *TATA‐box binding protein*. (B) Total protein was extracted from OVCAR4, OVCAR3 and Kuramochi cells and western blot analysis for CCDC80 and B7‐H3 was performed. GAPDH was used as a loading control. Blots shown are representative of three independent experiments. (C) Western blot analysis for CCDC80 in CCDC80‐overexpressing OVCAR3 and OVCAR4 cells or in Kuramochi cells 72 h following transfection with either NT‐siRNA or *CCDC80* siRNA (siCCDC80). Western blot for Cleaved Caspase‐3 was performed to assess apoptosis. β‐actin, GAPDH and Vinculin were used as loading controls. Blots shown are representative of three independent biological experiments. NT, non‐targeting. (D) MTS proliferation assay in stable CCDC80‐overexpressing OVCAR3 and OVCAR4 cells was performed 3 days after seeding or in Kuramochi cells 3‐ and 5‐days following CCDC80 knockdown. Data shown represent mean values ± standard error from three independent biological experiments, each performed with 6 technical replicates per condition. Statistical analysis was performed using a two‐tailed unpaired Student's *t*‐test **P < 0.01, ***P < 0.001. MTS ‐ 3‐(4,5‐dimethylthiazol‐2‐yl)‐5‐(3‐carboxymethoxyphenyl)‐2‐(4‐sulfophenyl)‐2H‐tetrazolium. (E) Colony formation assay in CCDC80‐overexpressing OVCAR4 or OVCAR3 cells. Experiments were performed in triplicates and representative images are shown. The number of colonies was calculated using ImageJ software. Graphs next to each image show the mean of three independent experiments. Error bars denote standard error. Paired Student's *t*‐test was used for statistical analysis, *P < 0.05. **P < 0.01. (F) Colony formation assay in Kuramochi cells with knockdown of PAX8, knockdown of CCDC80 or a knockdown of both. Representative images are shown on the left and a quantification of three independent experiments is shown on the right. Error bars denote standard error. ***P < 0.001, NS, not significant. (G) Control or CCDC80‐overexpressing OVCAR3 cells were injected subcutaneously into the flank of nude mice. Each group consisted of seven mice. Tumors were excised after 16 weeks and all resulting tumors are shown in the images. Quantification of the tumor numbers is shown on the right. The number of mice developing tumors in each group was compared using a one‐tailed Fisher's exact test. *P = 0.035.

Given that CCDC80 is negatively regulated by PAX8, we hypothesized that it may function as a tumor suppressor in HGSC. To test this hypothesis, we first analyzed CCDC80 expression in HGSC cell lines. As shown in Fig. 2A,B, Kuramochi HGSC cells express high levels of CCDC80 at both the mRNA and protein levels, while OVCAR3 and OVCAR4 cells exhibit significantly lower expression. To further explore the role of CCDC80 in HGSC, we stably overexpressed CCDC80 in OVCAR3 and OVCAR4 cells and knocked down its expression in Kuramochi cells (Fig. 2C). PAX8 has a pro‐proliferative and anti‐apoptotic role in HGSC (Fig. S1A,B), and therefore we sought to determine whether CCDC80, being negatively regulated by PAX8, could exert an opposing effect. Indeed, CCDC80‐overexpressing OVCAR3 and OVCAR4 cells exhibited a mildly reduced cell number compared to control vector cells. Conversely, CCDC80 knockdown in Kuramochi cells resulted in a modestly increased cell number at multiple time points (Fig. 2D). Furthermore, CCDC80 overexpression in OVCAR3 and OVCAR4 cells promoted apoptosis, as indicated by elevated levels of cleaved caspase‐3 (Fig. 2C). These findings suggest that the down regulation of CCDC80 may facilitate the pro‐proliferative and anti‐apoptotic effects of PAX8 in HGSC. However, the anti‐proliferative effect of CCDC80 is relatively minor, suggesting there are additional mediators of the pro‐proliferative role of PAX8, as we previously described [14, 26]. Similarly, PAX8 induces HGSC colony formation (Fig. S1C), and CCDC80 reduces HGSC colony formation (Fig. 2E), confirming another tumor suppressive property of CCDC80 in HGSC. When knocking down PAX8 and knocking down CCDC80, the number of colonies was restored to baseline (Fig. 2F), strengthening its role as a negative mediator of the role of PAX8 in HGSC. Based on the role of CCDC80 *in vitro* in proliferation and colony formation, we next tested its effect on tumor growth *in vivo*. CCDC80‐overexpressing and control OVCAR3 cells were injected subcutaneously into the flanks of seven nude mice each. Sixteen weeks later, tumors developed in four of seven mice injected with control OVCAR3 cells, whereas no tumors were observed in mice injected with CCDC80‐overexpressing cells (Fig. 2G and Fig. S1D), confirming CCDC80's role as a suppressor of tumor growth *in vivo*.

### 3.3 CCDC80 inhibits HGSC cell migration *in vitro* and *in vivo*

**Fig. 3:**
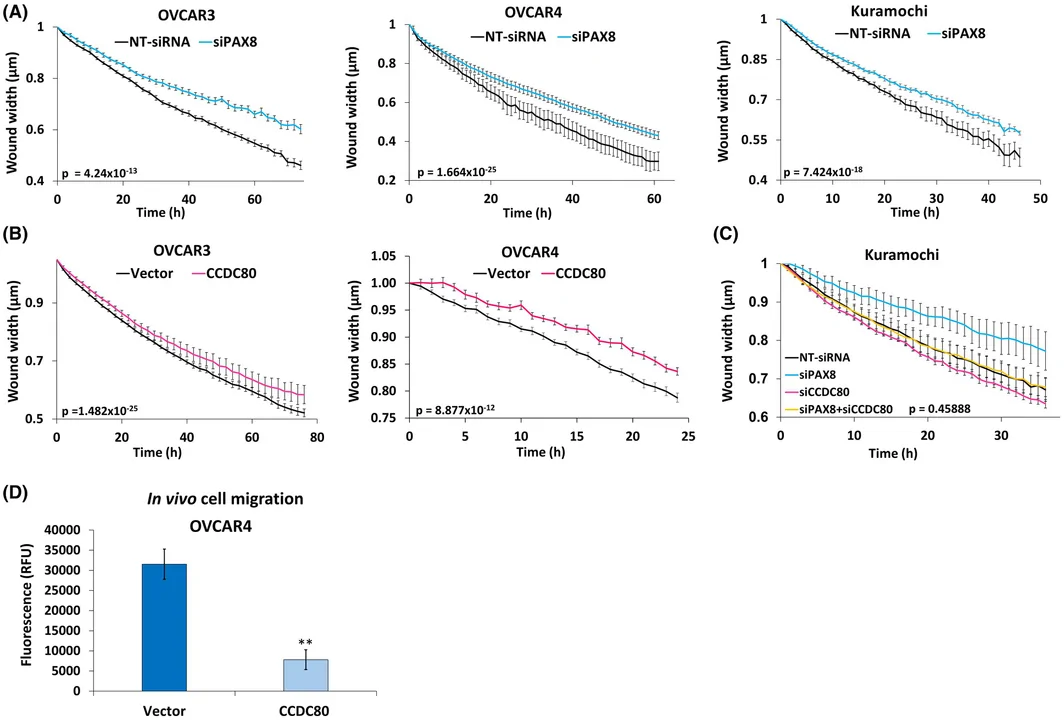
CCDC80 inhibits high‐grade serous ovarian cancer cell migration *in vitro* and *in vivo*. (A–C) Analysis of wound healing assay using the IncuCyte ZOOM™ real‐time live cell imaging system after the following transfections: (A) *PAX8* knockdown in OVCAR3, OVCAR4, and Kuramochi cells (OVCAR3: *n* = 6, OVCAR4, Kuramochi: *n* = 5). (B) *CCDC80* overexpression in OVCAR3 and OVCAR4 cells (OVCAR3: *n* = 5, OVCAR4: *n* = 20), or (C) transfection of Kuramochi cells with *siPAX8* alone, *siCCDC80* alone, or both (*n* = 5). Graph of the wound width over time is shown. Data shown represent mean values ± standard error. Paired Student's *t*‐test was used for statistical analysis. *P* value of *siPAX8* vs NT‐siRNA: 6.897 × 10^−16^; *P* value of *siCCDC80* vs *NT‐siRNA*: 4.816 × 10^−16^. The *P*‐value shown in the image is for NT‐siRNA compared to *siPAX8*+ *siCCDC80*. NT, non‐targeting. (D) Short‐term *in vivo* cell migration assay to the omentum. Fluorescently labeled CCDC80 OVCAR4 or Vector OVCAR4 cells were injected intraperitoneally to female nude mice and the omentum was harvested 24 h later, fluorescently labeled cancer cells were removed from the omentum, and fluorescence was measured using a plate reader. Data shown represent mean values ± standard error. *N* = 5 mice/group. Two‐tailed Student's *t*‐test was used for statistical analysis, **P < 0.01.

PAX8 has a positive effect on cell migration (Fig. 3A Fig. S2A) and we hypothesized that this role can also be mediated by the negative regulation of CCDC80. Therefore, we overexpressed CCDC80 in OVCAR3 or OVCAR4 cells and performed a wound healing assay. *CCDC80* overexpression led to slower cell migration *in vitro* (Fig. 3B and Fig. S2B), suggesting that CCDC80 has an antimigratory role in HGSC. To confirm that the inhibition of CCDC80 mediates the pro‐migratory role of PAX8, we knocked down PAX8 and knocked down CCDC80 to overcome its increased expression. The knockdown of CCDC80 reversed the positive effect of PAX8 on cell migration (Fig. 3C, Fig. S2C,D), suggesting that repression of CCDC80 can mediate the pro‐migratory effect of PAX8. Of note, to ensure that the effects observed in these experiments were due to cell migration rather than cell proliferation, we used low‐serum media to reduce proliferation and assessed migration at earlier time points than those used in our proliferation assays.

Finally, to establish CCDC80 as a tumor suppressor protein regulating HGSC cell migration and metastasis *in vivo*, we performed an *in vivo* cell migration assay. The most common site of HGSC metastasis is the omentum, and therefore we tested the ability of cancer cells to migrate and adhere to the omentum in mice. OVCAR4 HGSC cells stably expressing either CCDC80 or an empty vector were stained with a CMTPX fluorescent dye and injected intraperitoneally into female athymic nude mice. The omentum was harvested 24 h later, and fluorescent images of the omentum were taken by microscopy and quantified. Following this, the omentum was lysed, and fluorescent intensity of the lysates was measured by a plate reader device. As can be seen in Fig. 3D and Fig. S3, a marked decrease in omental metastasis was observed in mice injected with CCDC80 overexpressing cells compared to those injected with control cells, confirming the role of CCDC80 as an antimetastatic tumor suppressor in HGSC *in vivo*, reducing migration and adherence to the omentum.

### 3.4 CCDC80 negatively regulates B7‐H3 expression in HGSC

**Fig. 4:**
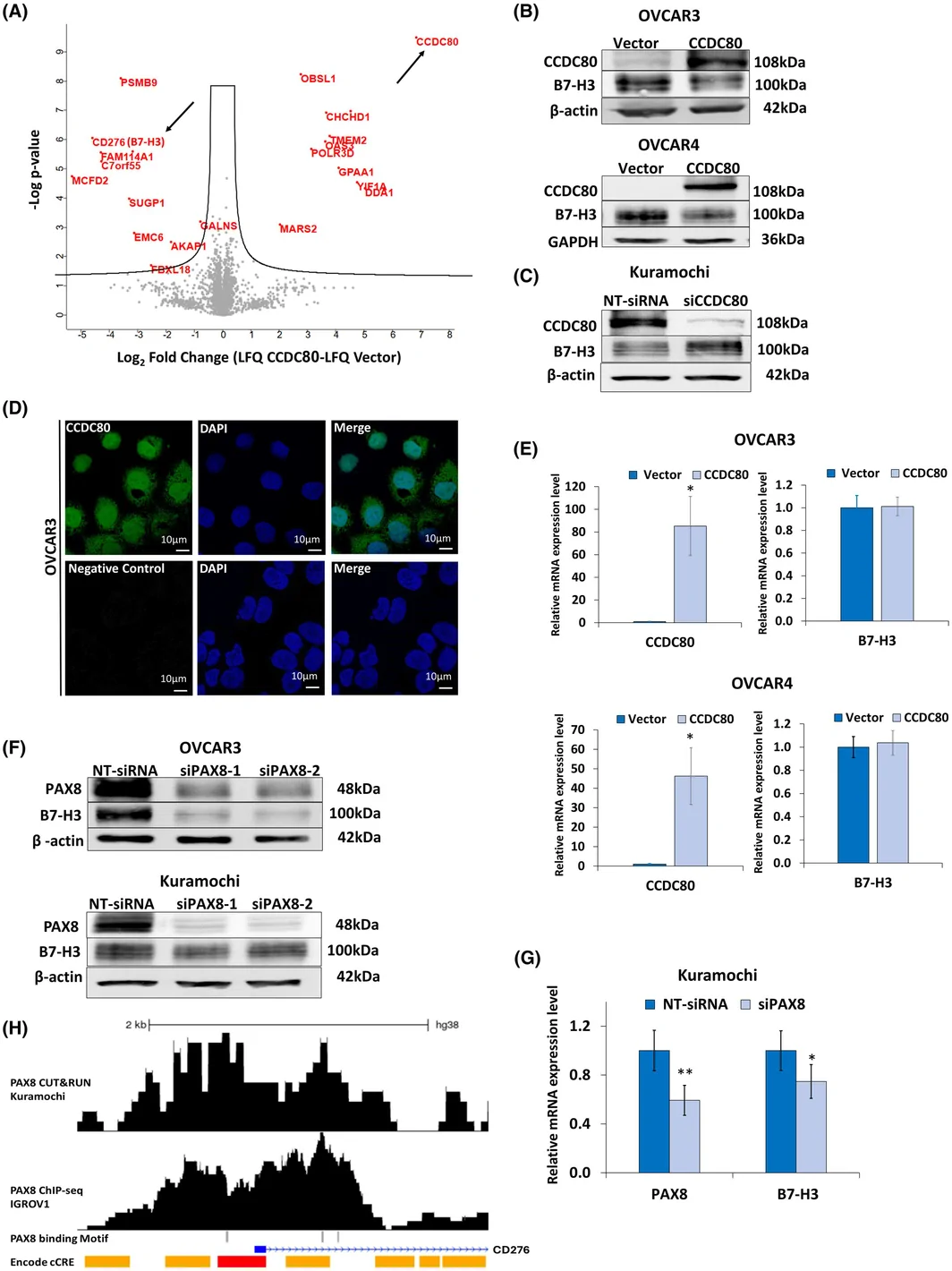
CCDC80 negatively regulates B7‐H3 expression in high‐grade serous ovarian cancer cells. (A) Volcano plot analysis of mass‐spectrometry results showing proteins significantly changed following CCDC80 overexpression in OVCAR4 cells. Shown are proteins increased or decreased by over 1.7‐fold change (log_2_ 0.8) with *P* value < 0.05. LFQ stands for label‐free quantification. Mass spectrometry analysis was performed from three independent biological samples per condition. LFQ ‐ label‐free quantification. (B, C) Western blot analysis for CCDC80 and B7‐H3 expression following CCDC80 overexpression in OVCAR3 and OVCAR4 cells (B) or CCDC80 knockdown in Kuramochi cells (C). GAPDH and β‐actin were used as loading controls. Blots shown are representative of three experiments. (D) Immunofluorescent staining for CCDC80 in OVCAR3 cells, 63× magnification, scale bar = 10 μm. Images are representative of three independent experiments. (E) *CCDC80* was overexpressed in OVCAR3 and OVCAR4 cells. Total RNA was extracted 24 h post transfection and mRNA expression level of *CCDC80* and *B7‐H3* was measured using real‐time qPCR. mRNA expression levels were normalized to *TBP* expression and shown relative to vector transfected cells. Data shown represent mean ± standard error from four independent experiments. *P < 0.05. Two‐tailed Student's *t*‐test was used for statistical analysis. qPCR, quantitative PCR. (F) PAX8 knockdown was performed in OVCAR3 and Kuramochi cells using two different siRNA constructs. Total protein was extracted 72 h later and western blot analysis for PAX8 and B7‐H3 was performed. β‐actin was used as a loading control. Blots shown are representative of three independent experiments. (G) *PAX8* was knocked down in Kuramochi cells. Total RNA was extracted 48 h post transfection and mRNA expression of *PAX8* and *B7‐H3* was quantified using real‐time qPCR. mRNA expression was normalized to *TBP* expression and shown relative to NT‐siRNA transfected cells. Data shown represent mean ± standard error from five independent experiments. Statistical analysis was performed using a two‐tailed Student's *t*‐test. * *P* < 0.05, **P < 0.01. NT, non‐targeting (H) PAX8 binding loci in the vicinity of the *CD276* (B7‐H3) transcription start site are shown at the two top rows. Third row shows predicted PAX8 binding sites, as analyzed by JASPAR. Bottom row shows candidate cis‐regulatory elements in the *CD276* locus based on ENCODE. The promoter is shown in red and enhancers in orange. ENCODE ‐ Encyclopedia of DNA Elements.

The biochemical function of CCDC80 is unknown, and therefore we wanted to study its role in an unbiased approach. Therefore, we overexpressed CCDC80 in OVCAR4 HGSC cells (Fig. S4) followed by mass spectrometry, comparing the results to vector transfected cells. Proteins that were differentially expressed with an over 1.7‐fold change between vector and CCDC80 transfected cells, and *P*‐value < 0.05, are listed in Table S4 and were highlighted in the volcano plot in Fig. 4A. As expected, CCDC80 was the most upregulated protein (Table S4). Among the differentially expressed proteins we observed a 24‐fold reduction in the immune checkpoint protein B7‐H3 (CD276) in CCDC80 overexpressing cells, making B7‐H3 the second most down‐regulated protein (Table S4). This finding was of particular interest, as B7‐H3‐targeting drugs are currently under evaluation in clinical trials [33]. B7‐H3 is expressed at comparable and detectable levels in OVCAR3, OVCAR4, and Kuramochi cells (Fig. 2B), and as expected overexpression of CCDC80 in OVCAR3 and OVCAR4 cells led to a decrease in B7‐H3 protein expression (Fig. 4B) and accordingly CCDC80 knockdown in Kuramochi cells led to B7‐H3 increase (Fig. 4C), confirming the negative regulation of B7‐H3 by CCDC80. We then asked what could be the mechanism by which CCDC80 represses B7‐H3 expression. Since the biochemical activity of CCDC80 is unclear, we thought we could get insight into its biochemical role by analyzing its subcellular localization. To test this, we performed immunofluorescent staining for CCDC80 in OVCAR3 and OVCAR4 cells and found that CCDC80 is mostly localized to the nucleus of HGSC cells (Fig. 4D and Fig. S5A). Despite the nuclear localization of CCDC80, we found that CCDC80 does not affect B7‐H3 mRNA levels (Fig. 4E), so the regulation of B7‐H3 is not transcriptional. Finally, since we have shown that PAX8 inhibits CCDC80 and CCDC80 inhibits B7‐H3 expression, we have shown that PAX8 upregulates B7‐H3 expression in different HGSC lines as expected (Fig. 4F and Fig. S5B). However, this regulation is not only mediated by CCDC80 since PAX8 positively regulates B7‐H3 mRNA expression (Fig. 4G), while CCDC80 does not (Fig. 4E). Although PAX8 was not bound to the *CD276* (B7‐H3) genomic region in our ChIP‐Seq data, we tested whether other PAX8 genomic localization studies found an interaction between PAX8 and B7‐H3. We queried a published CUT&RUN data studying the binding loci of PAX8 in Kuramochi HGSC cells [34]. This data shows PAX8 binding surrounding the *CD276* transcription start site. A similar PAX8 binding locus can be seen in a PAX8 ChIP‐seq of IGROV‐1 (GSM8417093), a non‐HGSC EOC line. Using JASPAR [35] we found three occurrences of the PAX8 binding element in the promoter and first intron of *CD276* (Fig. 4H and Fig. S5C). Taken together, these data support a plausible direct regulation of B7‐H3 by PAX8.

### 3.5 A cell autonomous role of B7‐H3 in HGSC

**Fig. 5:**
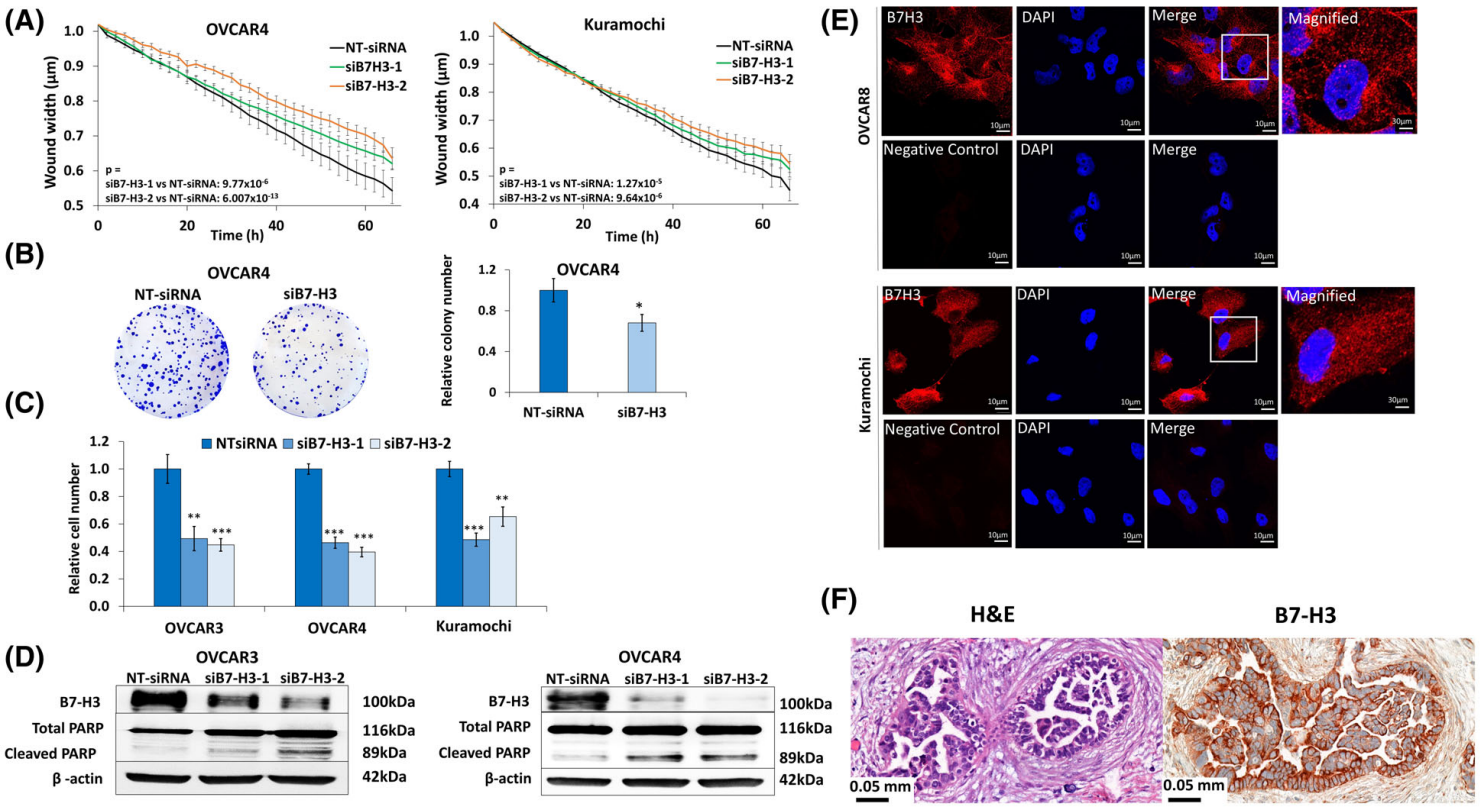
A cell autonomous role of B7‐H3 in high‐grade serous ovarian cancer. (A) Analysis of wound healing assay in OVCAR4 and Kuramochi HGSC cells following B7‐H3 knockdown with either NT‐siRNA or one of two different *B7‐H3* siRNA (siB7‐H3) constructs. Graph of wound width over time is shown. Data shown represent mean values (OVCAR4: *n* = 15, Kuramochi: *n* = 15). Statistical analysis was performed using paired Student's *t*‐tests. HGSC, high‐grade serous ovarian carcinoma, NT, non‐targeting. (B) Colony formation assay following B7‐H3 knockdown in OVCAR4 cells. Experiments were performed in triplicates and the number of colonies was calculated using ImageJ software. Figure shows a representative image and the mean of three independent experiments. Error bars denote standard error. Paired Student's *t*‐test was used for statistical analysis. *P < 0.05. (C) MTS cell proliferation assay in OVCAR3, OVCAR4 and Kuramochi cells 7 days post B7‐H3 knockdown using siRNA. Data shown represent mean values ± standard error from six independent experiments. Statistical comparisons were performed for each cell line between siB7‐H3–treated cells and NT‐siRNA–treated controls. Significance was determined using a two‐tailed, unpaired Student's *t*‐test. **P < 0.01, ***P < 0.001. MTS ‐ 3‐(4,5‐dimethylthiazol‐2‐yl)‐5‐(3‐carboxymethoxyphenyl)‐2‐(4‐sulfophenyl)‐2H‐tetrazolium. (D) B7‐H3 was knocked down in OVCAR3 and OVCAR4 cells. Total protein was extracted 48 h post transfection and western blot analysis for B7‐H3 and total and cleaved PARP was performed. β‐actin served as a loading control. Blots shown are representative of three independent experiments. (E) Immunofluorescent staining for B7‐H3 in OVCAR8 and Kuramochi cells, 63× magnification, scale bar = 10 μm. The rightmost images are a magnification of the squares in the images to their left with a scale bar = 30 μm. Images are representative of three independent experiments. (F) Immunohistochemical staining for B7‐H3 and H&E staining on paraffin embedded human high‐grade serous ovarian tumor tissue. 40× magnification, scale bar = 0.05 mm. Representative images from eight independent tumor samples are shown.

**Fig. 6:**
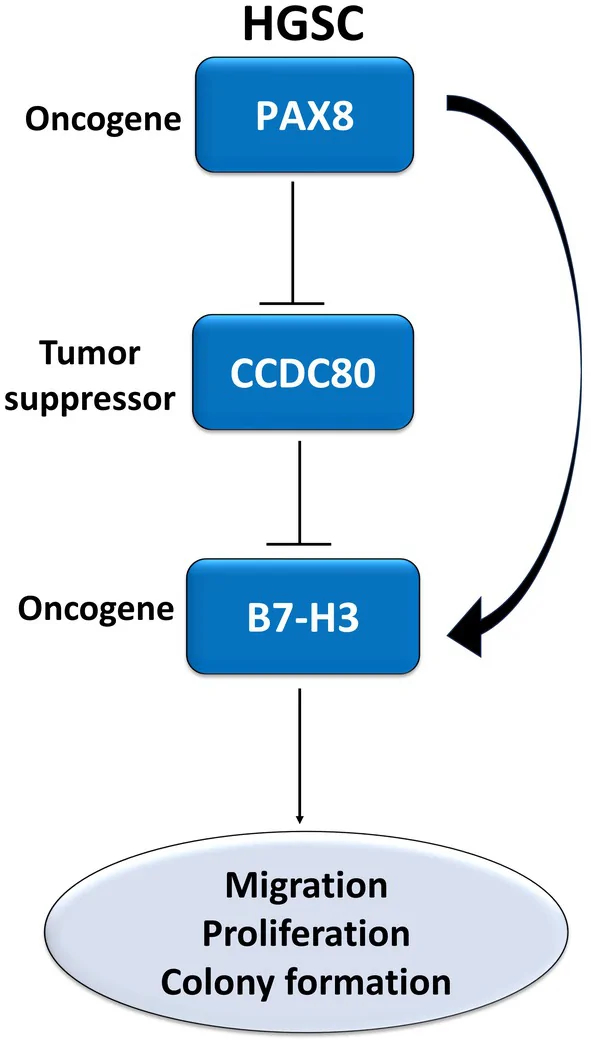
A proposed model for the cancer promoting activity of the PAX8/CCDC80/B7‐H3 signaling pathway in high‐grade serous carcinoma (HGSC). Illustration shows our proposed PAX8 signaling pathway in HGSC. PAX8 negatively regulates CCDC80 expression. CCDC80 inhibits B7‐H3 protein expression. In parallel, PAX8 positively regulates B7‐H3 mRNA expression in a CCDC80‐independent manner. This pathway promotes migration, proliferation, and colony formation in a cell‐autonomous manner.

B7‐H3 is mostly known as an immune checkpoint inhibitor that is expressed on cancer cells and regulates its interaction with stroma and immune cells [25]. However, there were other roles suggested for B7‐H3 such as a marker of cancer initiating cells [36], promoting migration and invasion [37, 38] and driving chemo resistance [37, 39]. Based on this, we hypothesized that B7‐H3 mediates the pro‐tumorigenic effects of PAX8 and the antitumorigenic effects of CCDC80 in a cell‐autonomous manner, independent of microenvironmental interactions. Our results confirmed that B7‐H3 has a cell autonomous promigratory role in HGSC (Fig. 5A, Fig. S5D for confirmation of the knockdown and Fig. S5E). Similarly, we showed that B7‐H3 enhances colony formation (Fig. 5B) and enhances cancer cell number (Fig. 5C) via inhibition of apoptosis (Fig. 5D). B7‐H3 is highly expressed by many human malignancies, making it an attractive immunotherapeutic target. However, its expression pattern in epithelial ovarian cancer has not been well‐characterized [40]. The TCGA data suggests that B7‐H3 expression is not correlated with survival [18] (Fig. S5F), but the TCGA uses bulk RNA and therefore cannot differentiate expression in cancer cells or the tumor microenvironment. However, it has been reported that most non‐immune cells in the EOC tumor microenvironment exhibited membranous staining for B7‐H3 [40]. We were interested in exploring the subcellular localization of B7‐H3 in the ovarian cancer cells to better understand its biochemical role and oncogenic activity in this disease. To assess the subcellular localization of B7‐H3, we stained HGSC cells for B7‐H3 (Fig. 5E), showing a clear cytoplasmic signal, although membranous staining could not be excluded. We then stained human HGSC tumors for B7‐H3. Seven out of eight tumors showed clear cytoplasmic staining, with four of these exhibiting both membranous and cytoplasmic expression (Fig. 5F shows a representative image with both localizations). B7‐H3 was not detected in the eighth sample. Taken together, we show here a cell autonomous oncogenic role for B7‐H3 in HGSC cell migration, colony formation, and evading apoptosis. Fig. 6 summarizes our proposed model for PAX8/CCDC80/B7‐H3 signaling pathway in driving the oncogenic capacities of PAX8 in HGSC.

## 4 Discussion

The concept of lineage dependency was first introduced in 2005 with the identification of the essential role of MITF in melanoma [41]. Also referred to as ‘lineage addiction’, this term describes a protein that is essential to promote the oncogenic properties of the cancer cell without acquiring an activating mutation or an increase in copy number. Usually, such lineage markers would have activity in cells of the same lineage during embryonic development or differentiation. Other known lineage dependencies are androgen receptor in prostate cancer, or FLT3 in myeloid malignancies [41]. Many of these lineage addiction proteins are transcription factors and master regulators of transcription in cancer, such as MITF, Androgen receptor, and PAX8, [10] and several studies show that lineage is the main determinant of genetic alterations across multiple tumor types [42, 43].

In this manuscript, we study the role of the lineage marker PAX8 in HGSC, the most common subtype of epithelial ovarian cancer. PAX8 has been shown to be a master transcriptional regulator with multiple oncogenic roles in HGSC [17, 19, 34]. Given its extensive oncogenic functions, and minimal expression in other tissue types, PAX8 has been proposed as a therapeutic target [16, 44, 45]. However, transcription factors are notoriously challenging drug targets [46], making it critical to understand the downstream signaling pathways regulated by PAX8 as a means of identifying alternative therapeutic targets. Identifying downstream effectors with multiple oncogenic functions that mediate distinct protumorigenic roles of PAX8 could be particularly valuable. Accordingly, this study focuses on downstream targets of PAX8, with an emphasis on CCDC80, which we identify as a novel tumor suppressor protein exhibiting multiple tumor‐suppressive properties in HGSC.

Interestingly, *CCDC80* is neither deleted nor mutated in HGSC [18]. A study looking at *CCDC80* expression levels in ovarian cancer showed its expression is reduced in ovarian cancer compared to other tissues, but this study used bulk quantitative real‐time PCR, which inherently cannot differentiate CCDC80 expression in cancer vs. stromal cells [24]. Most of the information regarding the role of CCDC80 in cancer is attributed to CCDC80 expressed in stromal cells. One very recent study showed that in normal fallopian tube tissue, CCDC80 is expressed in stromal and not in epithelial cells of the fallopian tube [47]. Furthermore, *CCDC80* knockout mice showed a propensity to develop ovarian cancer, but the tumors were a result of *CCDC80* deletion in the microenvironment rather than in cancer cells [22, 23]. Therefore, our cell line‐based *in vitro* system is the first to selectively study CCDC80 in epithelial HGSC cells, attributing a tumor suppressive role to epithelial CCDC80 as opposed to the stromal protein. We have shown a significantly reduced ability of HGSC cells overexpressing CCDC80 to form tumors, and to migrate and adhere to the omentum, hence metastasize *in vivo*. This role of CCDC80 in inhibiting HGSC cell migration aligns with the known function of other coiled‐coil domain proteins in suppressing migration, such as CCDC25 in renal cell carcinoma [48, 49]. CCDC25 inhibits cancer cell migration by interaction with neutrophil extracellular traps (NETs), a role that requires transmembrane expression [49]. CCDC170, another coiled‐coil domain containing protein, inhibits breast cancer cell migration via its expression in the Golgi apparatus [50]. We show that CCDC80 in HGSC tumors is expressed in the nuclei of cancer cells, suggesting that the mechanism of inhibition of migration is likely different from the mechanisms suggested here for other coiled‐coil domain proteins. We show that CCDC80 can affect cell migration via inhibition of B7‐H3 expression, but the mode of regulation is still unclear.

B7‐H3, also called CD276, is a transmembrane protein that exists in humans in two isoforms—2IgB7‐H3 and 4IgB7‐H3. The former isoform is composed of a single pair of IgV‐like and IgC‐like domains on the outer surface of the cells, and the latter contains two pairs. In normal cells B7‐H3 is expressed at low levels, mostly because it is downregulated by different miRNAs [51]. In cancer cells, B7‐H3 is expressed at high levels and plays a role in blocking adaptive immunity [52]. The role of B7‐H3 in promoting tumorigenesis, irrespective of the microenvironment, is less studied [36]. We show an important tumorigenic role for B7‐H3 that is unconnected to its role as an immune checkpoint protein. This different cell autonomous role could be connected to the unusual expression pattern of B7‐H3 in HGSC, in the cellular cytoplasm, and not just the membranous expression that is required for its immune regulating role. B7‐H3 is being targeted in the clinic by antibodies such as enoblituzumab [33], antibody drug conjugates such as Mirzotamab clezutoclax (ABBV‐155) [53], and radiolabeled antibodies such as ^131^I‐Omburtamab [54]. All the studies referred to B7‐H3 either as an immune checkpoint protein [33] or as a tumor marker used to target drugs or radioisotopes specifically to tumors [53, 54]. We propose that targeting these compounds by inhibitory antibodies can serve to directly suppress the tumorigenic properties of cancer cells.

## 5 Conclusions

In conclusion, we show a novel pathway that mediates the promigratory and protumorigenic properties of PAX8 in HGSC. We suggest CCDC80 as a novel tumor suppressor protein in HGSC that inhibits the ability of cancer cells to spread and metastasize and propose a new role for B7‐H3 in this disease. Targeting PAX8 or B7‐H3 in HGSC could be a way to prevent tumor growth and metastasis efficiently. Specifically, there are clinical grade antibodies targeting B7‐H3 that, to the best of our knowledge, have not been tested in HGSC. Our work supports adding this untreatable disease to the pipeline of B7‐H3 targeting clinical development.

## Conflict of interest

RP reports consulting fees or honoraria from Biond Biologics, 1E Therapeutics, Astra‐Zeneca, GSK, Neopharm, Simplivia and Genetech, and research funding to institution from 1E Therapeutics and Gilead Biosciences all unrelated to the current manuscript.

## Author contributions

AS was involved in conceptualization, acquisition of data, methodology, formal analysis, investigation, validation, visualization, writing (review and editing) and final approval of the manuscript. NMI was involved in acquisition of data, methodology, formal analysis and final approval of the manuscript. MC was involved in acquisition of data, methodology, formal analysis and final approval of the manuscript. EGS was involved in acquisition of data, methodology, formal analysis, and final approval of the manuscript. RGW was involved in acquisition of data, methodology, formal analysis, and final approval of the manuscript. IS was involved in acquisition of data, methodology, formal analysis, and final approval of the manuscript. IN was involved in acquisition of data, methodology, formal analysis, and final approval of the manuscript. HAK was involved in methodology and final approval of the manuscript. BF was involved in acquisition of data and final approval of the manuscript. LK was involved in acquisition of data and final approval of the manuscript. EG was involved in acquisition of data, methodology, formal analysis, and final approval of the manuscript. LB was involved in supervision, acquisition of data, analysis of data, drafting of manuscript, and final approval of the manuscript. RP was involved in supervision, conceptualization and design of study, interpretation and analysis of data, funding acquisition, resources, writing (original draft) and final approval of the manuscript.

## Supporting information


**Fig. S1.** PAX8 plays an anti‐apoptotic and pro‐clonogenic role in HGSC.
**Fig. S2.** PAX8 has a positive effect on cancer cell migration via inhibition of CCDC80.
**Fig. S3.** CCDC80 inhibits HGSC cell migration *in vivo*.
**Fig. S4.** Validation of CCDC80 overexpression in OVCAR4 cells.
**Fig. S5.** PAX8 regulates B7‐H3 expression, promoting cell migration.
**Table S1.** List of antibodies used in western blotting.
**Table S2.** List of primers used for real‐time qPCR.
**Table S3.** List of genes that are up or down regulated following PAX8 knockdown.
**Table S4.** Mass spectrometry results showing differentially expressed proteins in OVCAR4 cells following CCDC80 overexpression.

## Data Availability

RNA sequencing data of PAX8 knockdown in USPC can be found at the following URL: https://www.ncbi.nlm.nih.gov/sra/PRJNA727688. ChIP‐Seq raw data of PAX8 and H3K27Ac in HGSC can be found at the following URL: https://www.ncbi.nlm.nih.gov/sra/PRJNA1434028.
