# Supplementary material for: CCDC80 suppresses high‐grade serous ovarian cancer migration via negative regulation of B7‐H3
Source: Mol Oncol. 2026 Apr 21;20(9):2262–79. doi: 10.1002/1878-0261.70235 (PMC13398941; doi:10.1002/1878-0261.70235)
Supplement: Supplementary file 1 [file MOL2-20-2262-s001.docx]

Supplementary Tables

**Table S1****:** List of antibodies used in Western blotting.

| **Antibody** | **Manufacturer** | **Catalog number** |
| --- | --- | --- |
| PAX8 | Proteintech | 10336 |
| CCDC80 | R&D Systems | AF3410 |
| B7-H3 | Abcam | ab227670 |
| GAPDH | Santa Cruz Biotechnology | 32233 |
| β-actin | Cell Signaling Technology | 3700 |
| Vinculin | Abcam | ab129002 |
| PARP | Cell Signaling Technology | 9532S |
| Cleaved Caspase-3 | Cell Signaling Technology | 9664 |
| Donkey-anti-rabbit HRP | Jackson ImmunoResearch Labs | 1-035-154 |
| Bovine anti-goat HRP | Jackson ImmunoResearch Labs | 805-035-180 |
| Goat anti-mouse HRP | Jackson ImmunoResearch Labs | 115-035-003 |

**Table S2:** List of primers used for real-time qPCR

| **Gene** | **Primer sequence, 5'-3'‎** |
| --- | --- |
| *B7-H3* | F: GGCTTTCGTGTGCTGGAGAA |
|  | R: CTGTCAGAGTGTTTCAGAGG |
| *CCDC80* | F: CACGCAGAGTCCCAAGAAGT |
|  | R: GCCGAAGATGGTGATCACAG |
| *CTGF* | F: CAGGCTAGAGAAGCAGAGC |
|  | R: TGGTGCAGCCAGAAAGCTC |
| *FGF18* | F: GGACATGTGCAGGCTGGGCTA ‎ |
|  | R: GTAGAATTCCGTCTCCTTGCCCTT |
| *LMO3* | F: TCTGAGGCTCTTTGGTGTAACG |
|  | R: CCAGGTGGTAAACATTGTCCTTGG |
| *PAX8* | F: TCAACCTCCCTATGGACAGCTG |
|  | R: GAGCCCATTGATGGAGTAGGTG |
| *S100A1* | F: CGATGGAGACCCTCATCAAC |
|  | R: GTCTCCATTCTCGTCTAGCTC |
| *SHMT1* | F: TTGCCTCGGAGAATTTCGCC |
|  | R: GTCCCGCCATAGTATCTCTGG |
| *TBP* | F: GCCAGCTTCGGAGAGTTCTGGGATT |
|  | R: CGGGCACGAAGTGCAATGGTCTTTA |
| *TPM1* | F: AGTTGGATCGTGCCCAGGA |
|  | R: GTCGGCATCTTCAGCAATGTG |

**Table S3:** **List of genes that are up or down regulated following PAX8 knockdown.** Shown genes that were significantly differentially expressed in at least four out of the five cell lines used (3 HGSC and 2 USPC) with a false discovery rate <0.05.

*Fold Change (FC) values for USPC were calculated from published log_2_FC values. For negative log_2_FC values, a “minus” was added after calculating the FC to indicate a decrease in gene expression. Positive values indicate an increase in gene expression.

**Table S4:** **Mass spectrometry results showing differentially expressed proteins in OVCAR4 cells following CCDC80 overexpression.** Shown are proteins up or down regulated by fold change > 1.7 (log_2_ 0.8) with p values < 0.05.

Supplementary Figures

**Fig. S1.** **PAX8 plays an anti-apoptotic and pro-clonogenic role in high-grade serous cancer. (A)** OVCAR3, OVCAR4 and KURAMOCHI HGSC cells were seeded in 96-well plates and PAX8 was knocked down using siRNA. Cell proliferation was measured using MTS assay seven days post transfection. Data shown represent mean values ± standard error from six independent experiments, each performed with six technical replicates per condition. p value was calculated using unpaired Student's t-test, **p<0.01, ***p<0.001. **(B)** PAX8 was knocked down in OVCAR3 and OVCAR4 HGSC cells. Total protein was extracted 72hr post transfection and Western blot analyses for PAX8 and total and cleaved PARP were performed. GAPDH was used as a loading control. Blots shown are representative of three independent biological experiments. **(C)** Colony formation assay following PAX8 knockdown in OVCAR3 and OVCAR4 HGSC cells. Experiments were performed in triplicates and the number of colonies was calculated using ImageJ software. Figure shows a representative image and the mean of three independent experiments. Error bars denote standard error. Paired Student's t-test was used for statistical analysis. *p<0.05. **(D)** H&E stain of a representative OVCAR3 xenograft is shown at 20x magnification.

**Fig. S2. PAX8 has a positive effect on cancer cell migration via inhibition of CCDC80.**

**(A-C)** Wound healing images were captured by IncuCyte Zoom device at 10x magnification and 300μm scale bars. Treatments are shown to the left of each image, and time points are shown above each image. A representative image is shown for each experiment. **(A-B)** OVCAR3 cells. **(C)** Kuramochi cells. **(D)** Real-time mRNA expression quantification for validation of knockdown performed in Fig. 3C. *PAX8* mRNA expression is shown in the top graph and *CCDC80* mRNA expression on the bottom. All p values are compared to the NT-siRNA control. *p<0.05, ***p<0.001, NT – non-targeting, NS – non significant.

**Fig. S3. CCDC80 inhibits high-grade serous cancer cell migration *in vivo*.**

Short-term *in vivo* cell migration assay to the omentum. Fluorescently labeled CCDC80 OVCAR4 or Vector OVCAR4 cells were injected intraperitoneally to female nude mice, and the omentum was harvested 24 hours later. **(A)** Images of isolated omentum were taken using Olympus stereoscope. "No cells" represents the baseline fluorescence of the mouse omentum, without injected fluorescent cells. 63x magnification, scale bar - 2 mm **(B)** Quantification of the fluorescence intensity of omentum shown in the images. Data shown represent mean values ± standard error, five mice per group. One-tailed Student's t-test was used for statistical analysis, *p<0.05. Images here are the same experiment as Fig. 3D, but a different measurement method is used (fluorescence intensity).

**Fig. S4.** **Validation of CCDC80 overexpression in OVCAR4 cells.**

OVCAR4 cells were transfected with either an empty vector or a *CCDC80*-expressing vector in triplicates. Total protein was extracted 72hr later and Western blot analysis for CCDC80 was performed to confirm CCDC80 overexpression efficiency prior to mass spectrometric analysis. β-actin was used as a loading control.

**Fig. S5.** **PAX8 regulates B7-H3 expression - promoting cell migration.**

**(A)** Immunofluorescent staining for CCDC80 in OVCAR4 cells, 20x magnification, scale bar = 20 µm. Images are representative of three independent experiments. **(B)** Total protein was extracted from OVCAR8 cells 72hr post PAX8 knockdown and Western blot analysis for PAX8 and B7-H3 was performed. Vinculin was used as a loading control. Blots shown are representative of three independent experiments. **(C)** JASPAR logo showing PAX8 recognition element used in our analysis. **(D)** KURAMOCHI cells were transfected with either NT-siRNA or one of two different *B7-H3* siRNA (siB7-H3) constructs. Total protein was extracted 72 hours post transfection and a Western blot analysis for B7-H3 expression was performed for knockdown validation. Vinculin served as a loading control. Blots shown are representative of n three independent experiments. NT – non-targeting **(E)** Representative images of wound healing assay performed in OVCAR4 and KURAMOCHI cells following B7-H3 knockdown. Images were captured by an IncuCyte Zoom device at 10x magnification and 300 μm scale bars. Treatments are shown to the left of each image, and time points are shown above each image. **(F)** Analysis from TCGA showing that B7-H3 (*CD276*) expression does not have a prognostic value in high-grade serous ovarian cancer.
